# (1*S*,3a*S*,4*S*,7a*S*)-Ethyl 1-benzyl-2-(4-meth­oxy­benz­yl)-6,7-dimethyl-3-oxo-2,3,3a,4,5,7a-hexa­hydro-1*H*-isoindole-4-carboxyl­ate dichloro­methane monosolvate

**DOI:** 10.1107/S1600536813002560

**Published:** 2013-01-31

**Authors:** Benguo Lin, Jin-Long Wu

**Affiliations:** a Laboratory of Asymmetric Catalysis and Synthesis, Department of Chemistry, Zhejiang University, Hangzhou, Zhejiang 310027, People’s Republic of China

## Abstract

In the title compound, C_28_H_33_NO_4_·CH_2_Cl_2_, the pyrrolidone ring adopts a twisted envelope conformation and the cyclo­hexene has a half-chair conformation. In the crystal, weak C—H⋯O hydrogen bonds link the components into chains along [100].

## Related literature

For isoindolin-1-one derivatives in cytochalasins, see: Liu *et al.* (2006 ▶); Cox *et al.* (1983 ▶).
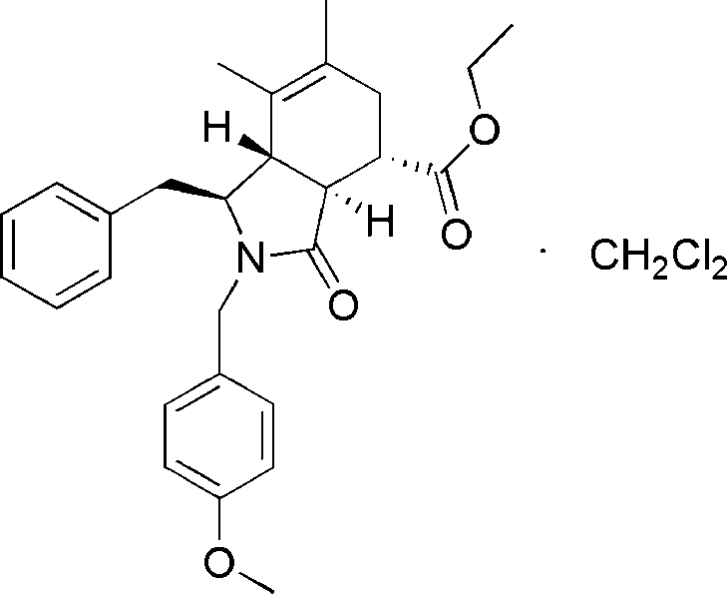


## Experimental

### 

#### Crystal data

C_28_H_33_NO_4_·CH_2_Cl_2_
*M* *_r_* = 532.48
Monoclinic, 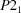

*a* = 9.6864 (5) Å
*b* = 15.5706 (8) Å
*c* = 9.7460 (6) Å
β = 109.221 (7)°
*V* = 1387.99 (14) Å^3^
*Z* = 2
Mo *K*α radiation
μ = 0.27 mm^−1^
*T* = 293 K
0.32 × 0.26 × 0.23 mm

#### Data collection

Oxford Diffraction Xcalibur (Atlas, Gemini ultra) diffractometer
Absorption correction: multi-scan (*CrysAlis PRO*; Oxford Diffraction, 2011 ▶) *T*_min_ = 0.919, *T*_max_ = 0.941
6260 measured reflections
3844 independent reflections
2990 reflections with *I* > 2σ(*I*)
*R*_int_ = 0.024

#### Refinement

*R*[*F* ^2^ > 2σ(*F* ^2^)] = 0.048
*wR*(*F* ^2^) = 0.127
*S* = 1.07
3844 reflections
330 parameters
1 restraint
H-atom parameters constrained
Δρ_max_ = 0.26 e Å^−3^
Δρ_min_ = −0.22 e Å^−3^
Absolute structure: Flack (1983 ▶), 1789 Friedel pairs
Flack parameter: −0.05 (11)

### 

Data collection: *CrysAlis PRO* (Oxford Diffraction, 2011 ▶); cell refinement: *CrysAlis PRO*; data reduction: *CrysAlis PRO*; program(s) used to solve structure: *SHELXS97* (Sheldrick, 2008 ▶); program(s) used to refine structure: *SHELXL97* (Sheldrick, 2008 ▶); molecular graphics: *OLEX2* (Dolomanov *et al.*, 2009 ▶); software used to prepare material for publication: *OLEX2*.

## Supplementary Material

Crystal structure: contains datablock(s) I, global. DOI: 10.1107/S1600536813002560/cv5380sup1.cif

Structure factors: contains datablock(s) I. DOI: 10.1107/S1600536813002560/cv5380Isup2.hkl

Supplementary material file. DOI: 10.1107/S1600536813002560/cv5380Isup3.cml

Additional supplementary materials:  crystallographic information; 3D view; checkCIF report

## Figures and Tables

**Table 1:** Hydrogen-bond geometry (Å, °)

*D*—H⋯*A*	*D*—H	H⋯*A*	*D*⋯*A*	*D*—H⋯*A*
C29—H29*B*⋯O2^i^	0.97	2.42	3.313 (7)	153
C6—H6⋯O3^ii^	0.93	2.65	3.562 (5)	166

Symmetry codes: (i) 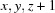; (ii) 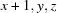.

**Fig. 1.**
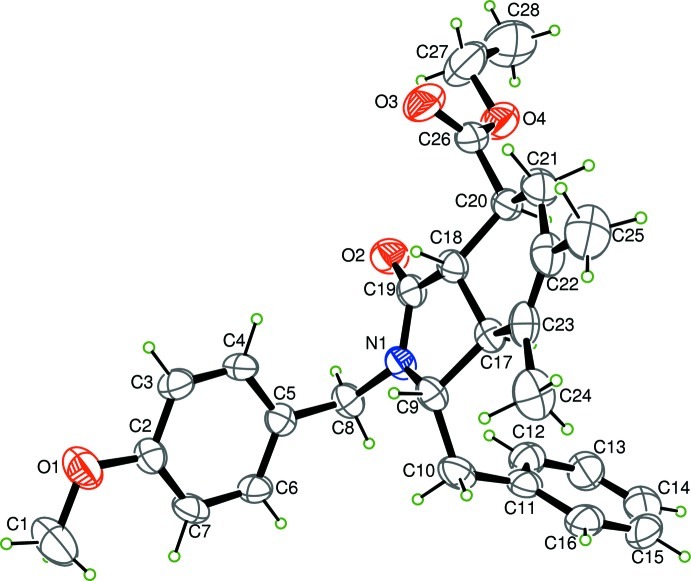
The molecular structure of the title compound with the atom numbering scheme. Displacement ellipsoids are drawn at the 40% probability level.

**Fig. 2.**
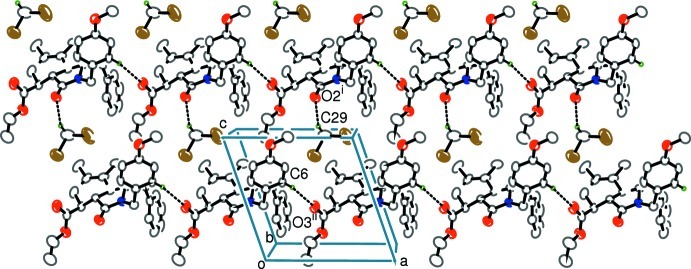
A portion of the crystal packing showing one-dimensional hydrogen-bonded (dashed line) chains [symmetry codes: (i) x, y, 1+z; (ii) 1+x, y, z].

## supplementary crystallographic information

### Comment

The scaffold of isoindolin-1-ones have derived many natural products and have great research values, such as cytochalasins (Liu *et al.* , 2006; Cox *et al.*, 1983). In our total synthesis of cytochalasin Z_8_, we have obtained the title compound as a major product through intramolecular Diels-Alder reaction. The structure of the title compound has been characterized by spectroscopic methods and further confirmation by X-ray analysis. The molecule has four rings including two benzene rings and one pyrrolidone ring and one cyclohexene ring and has four stereogenic centers as indicated absolute stereoconfiguration. We report here its crystal structure (Fig. 1). In the crystals of the title compound, the pyrrolidone ring C9/C17-C19/N1 adopts twisted envelope conformation, whereas the cyclohexene ring C17-C18/C20-C23 has a half chair conformation. The crystal packing (Fig. 2) is stabilized by two weak non-classical intermolecular C—H···O hydrogen bonds, the first one between H atom of dichloromethane and the carbonyl oxygen of the pyrrolidone, with a C29—H29B···O2^i^, and the second one between the H atom on the benzene ring of methoxybenzyl group and the carbonyl oxygen of the carboxylate group, with C6—H6···O3^ii^, respectively (Table 1).

### Experimental

To a solution of the (*S*,*E*)-*N*-(4-methoxybenzyl)-4,5-dimethyl-1-phenylhexa-3,5-dien-2-amine (272 mg, 0.85 mmol), 4-(dimethylamino)pyridine (DMAP) (10 mg, 0.085 mmol) and fumaric acid monoethyl ester (159 mg, 1.1 mmol) in dry CH_2_Cl_2_ cooled in an ice-water bath (273 K) under a nitrogen atmosphere, was added *N*, *N*'-diisopropylcarbodiimide (DIC) (164 mg, 1.3 mmol) and the reaction mixture was stirred for 4 hours at room temperature. After adding Celite, the mixture was stirred for another 30 minutes, and then the solid was filtered off and washed with CH_2_Cl_2_. The combined filtrate was evaporated under reduced pressure and the residue was purified by flash column chromatography (silica gel, 25% of ethyl acetate in hexane) to afford the title compound in 89% yield (338 mg) as a white solid (m.p. 381–382 K). Single crystals suitable for X-ray diffraction of the title compound were grown in CH_2_Cl_2_.

### Refinement

The H atoms were placed in calculated positions with C—H = 0.93– 0.98 Å according to their own hybridization model. And included in the refinement in riding model with *U*_iso_(H)=1.2*U*_eq_ or 1.5*U*_eq_ (sp^3^) of the carrier atom.

### Crystal data

**Table:**

C_28_H_33_NO_4_·CH_2_Cl_2_	*F*(000) = 564
*M**_r_* = 532.48	*D*_x_ = 1.274 Mg m^−^^3^
Monoclinic, *P*2_1_	Mo *K*α radiation, λ = 0.71073 Å
Hall symbol: P 2yb	Cell parameters from 2239 reflections
*a* = 9.6864 (5) Å	θ = 2.9–29.1°
*b* = 15.5706 (8) Å	µ = 0.27 mm^−^^1^
*c* = 9.7460 (6) Å	*T* = 293 K
β = 109.221 (7)°	Block, colourless
*V* = 1387.99 (14) Å^3^	0.32 × 0.26 × 0.23 mm
*Z* = 2	

### Data collection

**Table:**

Oxford Diffraction Xcalibur (Atlas, Gemini ultra) diffractometer	3844 independent reflections
Radiation source: fine-focus sealed tube	2990 reflections with *I* > 2σ(*I*)
Graphite monochromator	*R*_int_ = 0.024
Detector resolution: 10.3592 pixels mm^-1^	θ_max_ = 25.4°, θ_min_ = 2.9°
ω scans	*h* = −11→11
Absorption correction: multi-scan (*CrysAlis PRO*; Oxford Diffraction, 2011)	*k* = −18→14
*T*_min_ = 0.919, *T*_max_ = 0.941	*l* = −11→11
6260 measured reflections	

### Refinement

**Table:**

Refinement on *F*^2^	Hydrogen site location: inferred from neighbouring sites
Least-squares matrix: full	H-atom parameters constrained
*R*[*F*^2^ > 2σ(*F*^2^)] = 0.048	*w* = 1/[σ^2^(*F*_o_^2^) + (0.0493*P*)^2^ + 0.5082*P*] where *P* = (*F*_o_^2^ + 2*F*_c_^2^)/3
*wR*(*F*^2^) = 0.127	(Δ/σ)_max_ < 0.001
*S* = 1.07	Δρ_max_ = 0.26 e Å^−^^3^
3844 reflections	Δρ_min_ = −0.22 e Å^−^^3^
330 parameters	Extinction correction: *SHELXL97* (Sheldrick, 2008), Fc^*^=kFc[1+0.001xFc^2^λ^3^/sin(2θ)]^-1/4^
1 restraint	Extinction coefficient: 0.0155 (19)
Primary atom site location: structure-invariant direct methods	Absolute structure: Flack (1983), 1789 Friedel pairs
Secondary atom site location: difference Fourier map	Flack parameter: −0.05 (11)

### Special details

**Table:**

Geometry. All esds (except the esd in the dihedral angle between two l.s. planes) are estimated using the full covariance matrix. The cell esds are taken into account individually in the estimation of esds in distances, angles and torsion angles; correlations between esds in cell parameters are only used when they are defined by crystal symmetry. An approximate (isotropic) treatment of cell esds is used for estimating esds involving l.s. planes.
Refinement. Refinement of F^2^ against ALL reflections. The weighted R-factor wR and goodness of fit S are based on F^2^, conventional R-factors R are based on F, with F set to zero for negative F^2^. The threshold expression of F^2^ > 2sigma(F^2^) is used only for calculating R-factors(gt) etc. and is not relevant to the choice of reflections for refinement. R-factors based on F^2^ are statistically about twice as large as those based on F, and R- factors based on ALL data will be even larger.

### Fractional atomic coordinates and isotropic or equivalent isotropic displacement parameters (Å^2^)

**Table:**

	*x*	*y*	*z*	*U*_iso_*/*U*_eq_	
Cl1	0.9553 (2)	0.0250 (2)	1.0097 (3)	0.1866 (12)	
Cl2	0.65374 (18)	0.04546 (13)	0.85047 (17)	0.1164 (6)	
O1	1.3771 (3)	−0.2369 (2)	0.9604 (3)	0.0767 (10)	
O2	0.8238 (3)	−0.02886 (19)	0.3287 (3)	0.0632 (8)	
O3	0.5215 (3)	0.0189 (2)	0.4345 (4)	0.0860 (11)	
O4	0.5046 (3)	0.0707 (2)	0.2171 (3)	0.0750 (9)	
N1	1.0380 (3)	0.0314 (2)	0.4734 (3)	0.0468 (7)	
C1	1.5287 (6)	−0.2472 (4)	1.0238 (6)	0.0980 (19)	
H1A	1.5670	−0.2747	0.9558	0.147*	
H1C	1.5490	−0.2820	1.1095	0.147*	
H1B	1.5738	−0.1920	1.0495	0.147*	
C2	1.3277 (4)	−0.1863 (3)	0.8388 (4)	0.0495 (9)	
C3	1.1780 (4)	−0.1758 (3)	0.7830 (4)	0.0560 (10)	
H3	1.1186	−0.2016	0.8291	0.067*	
C4	1.1162 (4)	−0.1279 (3)	0.6604 (4)	0.0528 (10)	
H4	1.0151	−0.1221	0.6234	0.063*	
C5	1.2028 (4)	−0.0879 (2)	0.5907 (4)	0.0446 (9)	
C6	1.3511 (4)	−0.0988 (3)	0.6482 (4)	0.0497 (10)	
H6	1.4107	−0.0726	0.6028	0.060*	
C7	1.4150 (4)	−0.1476 (3)	0.7712 (4)	0.0540 (10)	
H7	1.5160	−0.1540	0.8077	0.065*	
C8	1.1352 (4)	−0.0356 (3)	0.4546 (4)	0.0524 (10)	
H8B	1.2124	−0.0094	0.4261	0.063*	
H8A	1.0805	−0.0736	0.3768	0.063*	
C9	1.0877 (4)	0.0985 (2)	0.5848 (4)	0.0464 (9)	
H9	1.1032	0.0723	0.6801	0.056*	
C10	1.2300 (4)	0.1407 (3)	0.5864 (5)	0.0587 (11)	
H10B	1.2683	0.1730	0.6761	0.070*	
H10A	1.2998	0.0956	0.5888	0.070*	
C11	1.2230 (4)	0.2007 (3)	0.4609 (5)	0.0541 (10)	
C12	1.1740 (4)	0.1739 (3)	0.3175 (5)	0.0594 (11)	
H12	1.1423	0.1176	0.2952	0.071*	
C13	1.1717 (5)	0.2301 (4)	0.2072 (6)	0.0779 (15)	
H13	1.1389	0.2109	0.1116	0.093*	
C14	1.2158 (5)	0.3122 (4)	0.2352 (8)	0.0866 (17)	
H14	1.2121	0.3493	0.1594	0.104*	
C15	1.2661 (5)	0.3406 (3)	0.3754 (8)	0.0840 (17)	
H15	1.2977	0.3971	0.3952	0.101*	
C16	1.2701 (4)	0.2854 (3)	0.4887 (6)	0.0705 (13)	
H16	1.3046	0.3053	0.5839	0.085*	
C17	0.9501 (4)	0.1545 (2)	0.5469 (4)	0.0426 (9)	
H17	0.9475	0.1889	0.4620	0.051*	
C18	0.8293 (4)	0.0886 (2)	0.4960 (4)	0.0455 (9)	
H18	0.8208	0.0606	0.5829	0.055*	
C19	0.8895 (4)	0.0240 (3)	0.4187 (4)	0.0470 (9)	
C20	0.6846 (4)	0.1320 (3)	0.4198 (4)	0.0515 (10)	
H20	0.6935	0.1668	0.3394	0.062*	
C21	0.6515 (5)	0.1908 (3)	0.5308 (5)	0.0699 (13)	
H21B	0.5880	0.2367	0.4785	0.084*	
H21A	0.5975	0.1580	0.5809	0.084*	
C22	0.7822 (5)	0.2308 (3)	0.6435 (5)	0.0604 (11)	
C23	0.9207 (5)	0.2151 (3)	0.6560 (4)	0.0556 (10)	
C24	1.0504 (6)	0.2564 (3)	0.7643 (5)	0.0743 (13)	
H24B	1.1009	0.2909	0.7143	0.111*	
H24C	1.1150	0.2129	0.8197	0.111*	
H24A	1.0185	0.2921	0.8284	0.111*	
C25	0.7356 (6)	0.2921 (3)	0.7405 (6)	0.0922 (18)	
H25B	0.6867	0.3406	0.6845	0.138*	
H25C	0.8202	0.3114	0.8176	0.138*	
H25A	0.6704	0.2632	0.7809	0.138*	
C26	0.5632 (4)	0.0670 (3)	0.3608 (5)	0.0645 (12)	
C27	0.3958 (7)	0.0046 (4)	0.1472 (7)	0.105 (2)	
H27B	0.4442	−0.0502	0.1491	0.126*	
H27A	0.3269	−0.0017	0.1997	0.126*	
C28	0.3196 (7)	0.0291 (5)	−0.0004 (7)	0.116 (2)	
H28C	0.3869	0.0304	−0.0539	0.173*	
H28B	0.2774	0.0850	−0.0020	0.173*	
H28A	0.2436	−0.0118	−0.0439	0.173*	
C29	0.7904 (7)	0.0628 (5)	1.0126 (7)	0.124 (2)	
H29A	0.7983	0.1239	1.0332	0.149*	
H29B	0.7654	0.0346	1.0900	0.149*	

### Atomic displacement parameters (Å^2^)

**Table:**

	*U*^11^	*U*^22^	*U*^33^	*U*^12^	*U*^13^	*U*^23^
Cl1	0.0957 (13)	0.250 (3)	0.191 (2)	−0.0212 (17)	0.0151 (13)	−0.069 (2)
Cl2	0.1087 (11)	0.1250 (14)	0.1006 (11)	−0.0138 (10)	0.0143 (9)	−0.0090 (10)
O1	0.064 (2)	0.099 (3)	0.069 (2)	0.0249 (18)	0.0239 (16)	0.0291 (19)
O2	0.0567 (17)	0.0626 (19)	0.0627 (18)	−0.0058 (15)	0.0091 (14)	−0.0102 (16)
O3	0.0700 (19)	0.094 (3)	0.096 (2)	−0.0166 (19)	0.0301 (18)	0.034 (2)
O4	0.0652 (18)	0.080 (2)	0.072 (2)	−0.0154 (17)	0.0118 (16)	0.0209 (17)
N1	0.0445 (17)	0.0443 (19)	0.0474 (17)	0.0077 (15)	0.0093 (13)	−0.0010 (15)
C1	0.079 (4)	0.128 (5)	0.079 (4)	0.039 (3)	0.016 (3)	0.029 (3)
C2	0.051 (2)	0.048 (2)	0.050 (2)	0.0079 (19)	0.0177 (18)	0.0022 (19)
C3	0.046 (2)	0.056 (3)	0.072 (3)	−0.006 (2)	0.027 (2)	0.002 (2)
C4	0.0315 (18)	0.053 (3)	0.073 (3)	−0.0017 (18)	0.0157 (19)	0.001 (2)
C5	0.047 (2)	0.039 (2)	0.047 (2)	0.0029 (17)	0.0149 (17)	−0.0075 (17)
C6	0.0374 (19)	0.054 (2)	0.062 (2)	−0.0011 (18)	0.0232 (18)	0.000 (2)
C7	0.0333 (18)	0.063 (3)	0.066 (3)	0.0107 (19)	0.0173 (18)	0.001 (2)
C8	0.053 (2)	0.050 (2)	0.053 (2)	0.009 (2)	0.0160 (19)	−0.0016 (19)
C9	0.046 (2)	0.049 (2)	0.038 (2)	0.0094 (18)	0.0060 (16)	−0.0004 (17)
C10	0.043 (2)	0.062 (3)	0.058 (3)	0.005 (2)	−0.0011 (18)	−0.011 (2)
C11	0.0334 (19)	0.054 (3)	0.077 (3)	0.0040 (18)	0.0197 (19)	−0.007 (2)
C12	0.055 (2)	0.059 (3)	0.067 (3)	−0.002 (2)	0.025 (2)	−0.006 (2)
C13	0.072 (3)	0.082 (4)	0.093 (4)	0.009 (3)	0.045 (3)	0.028 (3)
C14	0.064 (3)	0.085 (4)	0.134 (5)	0.019 (3)	0.064 (3)	0.038 (4)
C15	0.059 (3)	0.054 (3)	0.155 (6)	−0.003 (2)	0.057 (3)	0.007 (4)
C16	0.048 (3)	0.057 (3)	0.112 (4)	−0.002 (2)	0.033 (3)	−0.015 (3)
C17	0.048 (2)	0.047 (2)	0.0358 (19)	0.0074 (17)	0.0182 (16)	0.0057 (16)
C18	0.045 (2)	0.052 (2)	0.0399 (19)	0.0053 (18)	0.0133 (16)	0.0107 (17)
C19	0.048 (2)	0.048 (2)	0.0408 (19)	0.003 (2)	0.0095 (17)	0.0096 (19)
C20	0.043 (2)	0.057 (3)	0.057 (2)	0.0073 (19)	0.0209 (19)	0.021 (2)
C21	0.063 (3)	0.069 (3)	0.092 (3)	0.015 (2)	0.044 (3)	0.021 (3)
C22	0.081 (3)	0.051 (3)	0.067 (3)	0.018 (2)	0.048 (2)	0.008 (2)
C23	0.082 (3)	0.044 (2)	0.043 (2)	0.009 (2)	0.023 (2)	0.0101 (19)
C24	0.100 (4)	0.064 (3)	0.058 (3)	0.013 (3)	0.023 (3)	−0.017 (2)
C25	0.124 (4)	0.080 (4)	0.107 (4)	0.017 (3)	0.084 (4)	0.002 (3)
C26	0.043 (2)	0.067 (3)	0.084 (3)	0.004 (2)	0.023 (2)	0.029 (3)
C27	0.102 (4)	0.099 (4)	0.099 (4)	−0.044 (4)	0.013 (3)	0.021 (3)
C28	0.104 (4)	0.119 (5)	0.110 (5)	−0.030 (4)	0.015 (4)	−0.032 (4)
C29	0.132 (5)	0.144 (6)	0.085 (4)	0.014 (5)	0.020 (4)	−0.033 (4)

### Geometric parameters (Å, º)

**Table:**

Cl1—C29	1.712 (7)	C13—C14	1.347 (8)
Cl2—C29	1.716 (6)	C13—H13	0.9300
O1—C2	1.372 (5)	C14—C15	1.364 (7)
O1—C1	1.402 (6)	C14—H14	0.9300
O2—C19	1.218 (4)	C15—C16	1.389 (7)
O3—C26	1.197 (5)	C15—H15	0.9300
O4—C26	1.328 (5)	C16—H16	0.9300
O4—C27	1.470 (6)	C17—C18	1.511 (5)
N1—C19	1.364 (4)	C17—C23	1.517 (5)
N1—C8	1.456 (5)	C17—H17	0.9800
N1—C9	1.470 (5)	C18—C19	1.486 (5)
C1—H1A	0.9600	C18—C20	1.512 (5)
C1—H1C	0.9600	C18—H18	0.9800
C1—H1B	0.9600	C20—C26	1.513 (6)
C2—C7	1.371 (5)	C20—C21	1.531 (6)
C2—C3	1.381 (5)	C20—H20	0.9800
C3—C4	1.368 (5)	C21—C22	1.510 (7)
C3—H3	0.9300	C21—H21B	0.9700
C4—C5	1.389 (5)	C21—H21A	0.9700
C4—H4	0.9300	C22—C23	1.329 (6)
C5—C6	1.369 (5)	C22—C25	1.513 (6)
C5—C8	1.509 (5)	C23—C24	1.495 (6)
C6—C7	1.382 (5)	C24—H24B	0.9600
C6—H6	0.9300	C24—H24C	0.9600
C7—H7	0.9300	C24—H24A	0.9600
C8—H8B	0.9700	C25—H25B	0.9600
C8—H8A	0.9700	C25—H25C	0.9600
C9—C10	1.522 (5)	C25—H25A	0.9600
C9—C17	1.532 (5)	C27—C28	1.435 (8)
C9—H9	0.9800	C27—H27B	0.9700
C10—C11	1.522 (6)	C27—H27A	0.9700
C10—H10B	0.9700	C28—H28C	0.9600
C10—H10A	0.9700	C28—H28B	0.9600
C11—C12	1.385 (6)	C28—H28A	0.9600
C11—C16	1.394 (6)	C29—H29A	0.9700
C12—C13	1.380 (6)	C29—H29B	0.9700
C12—H12	0.9300		
			
C2—O1—C1	117.6 (4)	C18—C17—C9	102.2 (3)
C26—O4—C27	116.8 (4)	C23—C17—C9	122.3 (3)
C19—N1—C8	122.2 (3)	C18—C17—H17	107.0
C19—N1—C9	113.3 (3)	C23—C17—H17	107.0
C8—N1—C9	122.3 (3)	C9—C17—H17	107.0
O1—C1—H1A	109.5	C19—C18—C17	103.6 (3)
O1—C1—H1C	109.5	C19—C18—C20	120.5 (3)
H1A—C1—H1C	109.5	C17—C18—C20	110.6 (3)
O1—C1—H1B	109.5	C19—C18—H18	107.1
H1A—C1—H1B	109.5	C17—C18—H18	107.1
H1C—C1—H1B	109.5	C20—C18—H18	107.1
C7—C2—O1	125.0 (3)	O2—C19—N1	124.8 (4)
C7—C2—C3	119.5 (3)	O2—C19—C18	128.6 (3)
O1—C2—C3	115.5 (3)	N1—C19—C18	106.5 (3)
C4—C3—C2	120.6 (4)	C18—C20—C26	111.6 (3)
C4—C3—H3	119.7	C18—C20—C21	107.2 (3)
C2—C3—H3	119.7	C26—C20—C21	110.6 (3)
C3—C4—C5	120.7 (3)	C18—C20—H20	109.1
C3—C4—H4	119.6	C26—C20—H20	109.1
C5—C4—H4	119.6	C21—C20—H20	109.1
C6—C5—C4	117.7 (3)	C22—C21—C20	116.1 (3)
C6—C5—C8	121.3 (3)	C22—C21—H21B	108.3
C4—C5—C8	120.9 (3)	C20—C21—H21B	108.3
C5—C6—C7	122.2 (3)	C22—C21—H21A	108.3
C5—C6—H6	118.9	C20—C21—H21A	108.3
C7—C6—H6	118.9	H21B—C21—H21A	107.4
C2—C7—C6	119.2 (3)	C23—C22—C21	124.7 (4)
C2—C7—H7	120.4	C23—C22—C25	124.0 (5)
C6—C7—H7	120.4	C21—C22—C25	111.3 (4)
N1—C8—C5	112.8 (3)	C22—C23—C24	125.0 (4)
N1—C8—H8B	109.0	C22—C23—C17	117.8 (4)
C5—C8—H8B	109.0	C24—C23—C17	117.1 (4)
N1—C8—H8A	109.0	C23—C24—H24B	109.5
C5—C8—H8A	109.0	C23—C24—H24C	109.5
H8B—C8—H8A	107.8	H24B—C24—H24C	109.5
N1—C9—C10	113.0 (3)	C23—C24—H24A	109.5
N1—C9—C17	100.3 (3)	H24B—C24—H24A	109.5
C10—C9—C17	118.0 (3)	H24C—C24—H24A	109.5
N1—C9—H9	108.3	C22—C25—H25B	109.5
C10—C9—H9	108.3	C22—C25—H25C	109.5
C17—C9—H9	108.3	H25B—C25—H25C	109.5
C11—C10—C9	117.1 (3)	C22—C25—H25A	109.5
C11—C10—H10B	108.0	H25B—C25—H25A	109.5
C9—C10—H10B	108.0	H25C—C25—H25A	109.5
C11—C10—H10A	108.0	O3—C26—O4	123.2 (4)
C9—C10—H10A	108.0	O3—C26—C20	124.3 (4)
H10B—C10—H10A	107.3	O4—C26—C20	112.4 (4)
C12—C11—C16	117.6 (4)	C28—C27—O4	109.4 (5)
C12—C11—C10	122.4 (4)	C28—C27—H27B	109.8
C16—C11—C10	120.0 (4)	O4—C27—H27B	109.8
C13—C12—C11	120.5 (5)	C28—C27—H27A	109.8
C13—C12—H12	119.8	O4—C27—H27A	109.8
C11—C12—H12	119.8	H27B—C27—H27A	108.2
C14—C13—C12	121.4 (6)	C27—C28—H28C	109.5
C14—C13—H13	119.3	C27—C28—H28B	109.5
C12—C13—H13	119.3	H28C—C28—H28B	109.5
C13—C14—C15	119.7 (5)	C27—C28—H28A	109.5
C13—C14—H14	120.1	H28C—C28—H28A	109.5
C15—C14—H14	120.1	H28B—C28—H28A	109.5
C14—C15—C16	120.2 (5)	Cl1—C29—Cl2	111.8 (4)
C14—C15—H15	119.9	Cl1—C29—H29A	109.3
C16—C15—H15	119.9	Cl2—C29—H29A	109.3
C15—C16—C11	120.6 (5)	Cl1—C29—H29B	109.3
C15—C16—H16	119.7	Cl2—C29—H29B	109.3
C11—C16—H16	119.7	H29A—C29—H29B	107.9
C18—C17—C23	110.4 (3)		

### Hydrogen-bond geometry (Å, º)

**Table:**

*D*—H···*A*	*D*—H	H···*A*	*D*···*A*	*D*—H···*A*
C29—H29*B*···O2^i^	0.97	2.42	3.313 (7)	153
C6—H6···O3^ii^	0.93	2.65	3.562 (5)	166

Symmetry codes: (i) *x*, *y*, *z*+1; (ii) *x*+1, *y*, *z*.

## References

[bb1] Cox, R. H., Cutler, H. G., Hurd, R. E. & Cole, R. J. (1983). *J. Agric. Food Chem.* **31**, 405-408.

[bb2] Dolomanov, O. V., Bourhis, L. J., Gildea, R. J., Howard, J. A. K. & Puschmann, H. (2009). *J. Appl. Cryst.* **42**, 339–341.

[bb3] Flack, H. D. (1983). *Acta Cryst.* A**39**, 876–881.

[bb4] Liu, R., Gu, Q., Zhu, W., Cui, C., Fan, G., Fang, Y., Zhu, T. & Liu, H. (2006). *J. Nat. Prod.* **69**, 871–875. 10.1021/np050201m 16792402

[bb5] Oxford Diffraction (2011). *CrysAlis PRO* Oxford Diffraction Ltd, Yarnton, England.

[bb6] Sheldrick, G. M. (2008). *Acta Cryst.* A**64**, 112–122. 10.1107/S0108767307043930 18156677

